# High versus Low-Moderate Intensity Exercise Training Program as an Adjunct to Antihypertensive Medication: A Pilot Clinical Study

**DOI:** 10.3390/jpm11040291

**Published:** 2021-04-10

**Authors:** Vicente Ávila-Gandía, Maravillas Sánchez-Macarro, Antonio Luque-Rubia, Esther García-Sánchez, Fernando Cánovas, Asensio López-Santiago, Francisco Javier López-Román

**Affiliations:** 1 Department of Exercise Physiology, Universidad Católica de Murcia, 30107 Murcia, Spain; msanchez4@ucam.edu (M.S.-M.); ajluque@ucam.edu (A.L.-R.); fcanovas@ucam.edu (F.C.); jlroman@ucam.edu (F.J.L.-R.); 2 Fundación para la Formación e Invetigación Sanitarias de la Región de Murcia, 30003 Murcia, Spain; esther.garcia11@carm.es; 3 Servicio Murciano de Salud, 30120 Murcia, Spain; asensio.lopez@carm.es; 4 Primary Care Research GroupBiomedical Research Institute of Murcia (IMIB–Arrixaca), 30120 Murcia, Spain

**Keywords:** blood pressure monitoring, hypertension, physical exercises, primary care, training intensity

## Abstract

Objective: In this pilot clinical study we investigated the effect on blood pressure (BP) of two community-based exercise training programs of high (HIT) vs. low-moderate intensity (LMIT) in hypertensive individuals receiving at least one antihypertensive drug. Methods: The study included two phases of physical exercises based on 1-h session, 3 days/week for 12 and 16 weeks, respectively, separately by a 7-week resting period. Each phase was preceded by a four-week conditioning training period. According to the average maximal heart rate at baseline, participants were randomized to HIT (80–90%), LMIT (50–70%) or no-exercise (control). Heart rate was monitored during workout and BP profiles were registered by ambulatory BP monitoring at the beginning and end of each phase. Results: Of 60 individuals randomized, 44 completed the study (HIT, *n* = 10; LMIT, *n* = 16; controls, *n* = 18). BP levels were significantly reduced after the second phase for both LMIT (SBP −3.1 mmHg, DBP −2.4 mmHg) and HIT (SBP −10.8 mmHg, DBP −8.3 mmHg). Similar levels of improvement were also found in daytime and night-time BP. Mean attendance of the prescribed training sessions was 87.4 ± 6.2% for HIT and 87.4 ± 5.3% for LMIT during the first phase and 84.1 ± 5.0% and 85.2 ± 5.9% during the second phase, respectively (*p* = 0.047). Conclusion: Both HIT and LMIT exercise training programs reduced BP but the HIT modality showed a lower rate of compliance with proposed training schedule. Intensity of training should be individually prescribed to improve tolerance to more high intensity exercises.

## 1. Introduction

Lifestyle modifications with weight-reducing diet, regular exercise, and restrict salt and alcohol intake are key strategies in the prevention and management of hypertension for the goal of reducing cardiovascular morbidity and mortality [1]. In adults with hypertension, different types of physical exercise training programs are able to reduce blood pressure (BP) [2,3], particularly resistance exercise training to reduce diastolic BP (DBP) [2,4]. Combined aerobic and resistance training are also effective to reduce BP in hypertensive adults [5,6], and endurance training has been shown to be successful in decreasing resting systolic BP (SBP) and DPB [7]. Although the mechanisms of the effects of exercise training are complex and not fully understood, emerging evidences suggest a relationship with the intensity of training (low-to-moderate vs. high) [8,9,10]. There is consensus regarding benefits and effectiveness of moderate or even less intensity exercise training [9,10]. However, high intensity interval training could lead to physiological adaptations that would contribute to reduce the risk of high BP [8].

Community-based interventions to promote physical activity in the general population have been a focus of increasing interest [11,12], and the European Society of Cardiology (ESC) and the European Society of Hypertension (ESH) [13] recommend a moderate intensity aerobic training for at least 30 min on most (preferably all) days of the week. However, there are still unsolved questions regarding the best type of exercise to be prescribed (e.g., aerobic, or resistance) and the adequate percentage of intensity about the maximum in order to obtain optimal health benefits [14]. Moreover, data are still unclear whether benefits are due to a higher volume of energy expended or as a result of a vigorous-intensity exercise training, although there are supporting evidences for the latter [15].

In a previous study, we have shown the feasibility and benefits of implementing a preventive physical exercise program prescribed by primary care physicians in cardiovascular risk patients, with improvements in anthropometric and fitness-related variables [16]. However, the effects on BP in the population of hypertensive patients were not evaluated. The present pilot study was designed to assess the effect on BP of two community-based exercise training programs of high (HIT) vs. low-moderate intensity (LMIT) in hypertensive individuals receiving at least one antihypertensive drug and without any previous history of regular physical training. Variations in BP were determined by ambulatory BP monitoring (ABPM) due to the design of two modalities of physical exercise programs, in which the intensity of training was the only variable at the same amount of volume of energy expended.

## 2. Materials and Methods

### 2.1. Study Design and Population

Between January 2016 and December 2019, a pilot clinical study was conducted at the Health Sciences Department of the Saint Anthony Catholic University in Murcia, Spain. The primary objective of the study was to assess the effects of an exercise training program intervention of different intensities (high intensity vs. low-moderate intensity) on lowering BP as an adjunct strategy in hypertensive individuals under treatment with at least one antihypertensive drug. All participants included in the study were referred by their primary care physicians who prescribed physical exercise as a healthy lifestyle intervention added to antihypertensive regimens in the framework of the “ACTIVA-Murcia Program” [16], which advocates for citizens involvement in health care by promoting behavioral and lifestyle healthy habits.

Eligible participants were males and females, aged 40 to 65 years old, diagnosed with hypertension, following an antihypertensive therapy with at least one drug of over 1-year treatment without changes in the prescription and without history of regular physical training. Exclusion criteria were severe or terminal illness, diagnosis of ischemic heart disease and/or cerebrovascular disease or any cardiovascular disease (e.g., peripheral artery disease), diseases that would limit the execution of aerobic or resistance exercises (musculoskeletal disorders, restrictive lung disease, arrhythmia), diabetes mellitus, severe mental disorder, pregnant or breast-feeding women, and incapacity to understand the informed consent.

The study protocol was approved by the Ethics Committee of Saint Anthony Catholic University, Murcia (Spain). All participants gave written informed consent.

### 2.2. Physical Training Program

The same exercise training program was followed by all participants, with the only difference being the intensity of training. The program included two phases of physical exercises of 12 weeks (from 20 September 2016 to 23 December 2016) and 16 weeks (from 4 February 2017 to 12 June 2017) duration, respectively, separated by a 7-week resting period, which was coincident with a vacation period. The physical training program was carried out 3 days a week in 1-h sessions. Minimum compliance was considered, estimating attendance of 66% of the sessions as suitable threshold for completion of the training program. Each participant was randomized to one of three study groups of high intensity training (HIT), low-moderate intensity training (LMIT) or no-training (control) in a 1:1:1 ratio using Epidata software version 4.0, (EpiData Association, Odense, Denmark).

The intensity of training was established at one baseline session of aerobic exercise testing on a treadmill following a modification of the Balke–Ware protocol [17] but using the same protocol for men and women [18]. Warm up exercise was developed previous to the test during 2 min: first minute at a speed of 3 km/h and 1.5% slope, and second minute at 4 km/h and 4% slope. Test was divided into 15 phases of 1 min each with increments of speed (0.2 km/h) and slope (1%), starting at a speed of 5 km/h and 5% slope. All participants were monitored by ECG and gas analyzer (Jaeger Oxicom Pro^®^, Jaeger, Wuerzburg, Germany) in order to determine the maximum heart rate (MHR) and monitor heart rate (HR) above anaerobic threshold and during maximal oxygen uptake (VO2 max).

The physical training program was carried out 3 days a week in 1-h sessions focused on basic exercises of endurance (Global bodily activities), strength (Activities specific muscle regions) and flexibility. Physical activity was based on standardized fitness and wellness training and adapted to hypertensive patients by reducing intensity and applying restriction of movements. The program was developed by graduates in Sciences of Physical Activity and Sports, who were responsible for teaching the exercises and supervised every training session. Sessions were structured in blocks that provided a dynamic combination of exercises, avoiding an extended process of routine learning. As shown in Table 1, each session included a warm up with muscular activation, articular mobility and stretching exercises; an aerobic exercise interval at the desired HR target according to MHR recorded at the baseline aerobic exercise testing; and a final cool down based on stretching exercises. HR was monitored using a pulsometer (Polar RS400, Polar Electro Oy, Kempele, Finland) and loads of the exercises were targeted at 80–90% of MHR for the HIT group and at 50–70% of HR for the LMIT group. HR recordings were stored and analyzed for later assessment of adherence to intensity of the corresponding training program. Participants assigned to the HIT and LMIT groups received guidelines to continue physical exercise during the 7-week resting period, whereas participants in the control group were offered participation in the program after the study.

**Figure 1:**
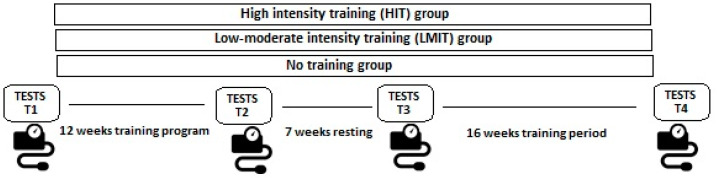
Participants were assigned to HIT, LMIT or no training groups, and the program included a first 12-week training phase followed by a 7-week resting period, and a 16-week training phase.

The course of the study is shown in Figure 1.

### 2.3. Data Collection

BP profiles were assessed by ABPM and recorded at the beginning (time #1, T1) and at the end of the first 12-week intervention phase of the study (time #2, T2), at week 19 after the 7-week resting period and prior to the beginning the second 16-week intervention phase (time #3, T3) and at week 35 at the end of the study (time #4, T4). Forty-eight hours after the last training sessions for every cited period, each patient was given a 24-h oscilometric blood pressure monitor device (Spacelabs Healthcare ABP monitor model 90217A, Spacelabs Medical Inc., Redmond, WA, USA) with the cuff width encircling 40% of the arm circumference and cuff length at least 80–100%. BP was measured at intervals of 30 min during the day and 60 min at night. Participants filled a logbook about relevant aspects of daily life (hours of labor, diet, sleeping hours, and habits) in order to synchronize BP monitor records with day activity and resting time. Night-time period was considered from 22:00 p.m. to 6:00 a.m. An entry was only considered valid when more than 70% of data were properly recorded, adding a minimum of 20 daytime readings and 7 night-time readings. Observations were then summarized as mean and standard deviation (±SD) as well as the difference of increment from baseline for systolic BP (SBP), diastolic BP (DBP), and pulse pressure (PP) during daytime, night-time and overall. Mean arterial pressure (MAP) was calculated as DBP + 1/3 PP. Systolic and diastolic load were estimated as the percentage of BP readings above reference values of SBP and DBP, respectively [13].

### 2.4. Statistical Analysis

The analysis of variance (ANOVA) for repeated measures was used to compare BP variables derived from ABPM profiles that were obtained at the beginning and end of the two defined phases of the trial. All variables were checked for normality. Homogeneity of variables among groups was also checked at baseline in order to avoid confounder variables. Training intensity (HIT, LMIT, and control) was included as an inter-subject factor in the analyses. Bonferroni correction was used for all comparisons between intervention groups and control. Type I error rate was set at α = 0.05. The per-protocol (PP) data set was analyzed. Analyses were also performed in the intention-to-treat (ITT) population. The SPSS statistical software v.21.0 (IBM Corp., Armonk, NY, USA) was used for the analysis of data.

## 3. Results

**Figure 2:**
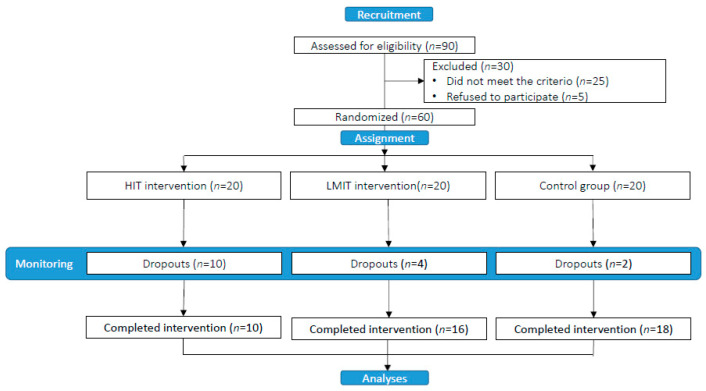
Flow chart of the study population.

A total of 90 subjects were assessed for eligibility, 25 of whom did not meet the inclusion criteria and 5 refused to participate. The study population included 60 hypertensive subjects, 20 of which were randomly assigned to each study group. However, 44 (73.3%) participants (19 men, 25 women; mean age 56.4 years) completed the study (minimum compliance of 66% of training sessions during the programmed sessions) (HIT, *n* = 10; LMIT, *n* = 16; controls, *n* = 18). The distribution by gender (females) showed no significant differences (chi-square test, *p* = 0.814). The flow chart of the study population is shown in Figure 2.

There were no statistically significant differences in age, BMI, lean body mass, fat body mass, SBP, and DBP when baseline values and values obtained at the end of the study were compared for either within-group or between-group differences (Table 2).

Anti-hypertensive pharmacological treatment included angiotensin receptor blockers in 42.5%, diuretics in 23.4%, beta-blockers in 14.9%, calcium antagonists in 10.6%, angiotensin-converting enzyme inhibitors in 6.4%, and alpha-blockers in 2.2%. In two patients assigned to the HIT group, the dose of beta-blockers was reduced by their primary care physician during the study. Differences in anti-hypertensive treatment by groups were not found (Table 3).

No statistically significant differences in 24-h ABPM variables for any group between the beginning (T1) and the end (T2) of the first 12-week phase of the study were observed (Table 3). During the second intervention phase of the study, mean decreases in SBP (−3.1 mmHg, 95% CI −6.7 to 0.6), DBP (−2.4 mmHg, 95% CI −4.4 to −0.5), systolic load (−6.2%), and diastolic load (−8.6%) observed in the LMIT were not statistically significant. However, all these changes in the HIT group were significant (*p* < 0.001), with mean decreases of SBP of −10.8 mmHg (95% CI −15.4 to −6.2), DBP of −8.3 mmHg (95% CI −10.8 to −5.8), systolic load of −27.0%, and diastolic load of −26.9% (Table 4). Similar results were obtained in the ITT analysis (see Table S1 in the Supplementary Materials).

Changes in daytime AMBP variables showed a similar profile than 24-h recordings (Table 5), with statistically significant differences in decreases of average SBP (−11.2 mmHg), average DBP (−8.2 mmHg), MAP (−9.2 mmHg), systolic load (−24.4%), and diastolic load (−27.3%) in the second phase of the study for the HIT group only. In the night-time AMBP recordings (Table 6), similar findings were found in the HIT group, with significant reductions of average SBP (−9.5 mmHg), DBP (−7.0 mmHg), MAP (−7.8 mmHg), systolic load (−35.9%), and diastolic load (−26.6%) during the second phase of the study. Differences between beginning of the first phase of the study and at the study end were also observed in average SBP (−7.4 mmHg) and MAP (−6.1 mmHg).

Average compliance was 86% of training sessions (range 79% to 100%). During the first 12-week phase of the study, subjects attended to 87.4 ± 5.3% of LMIT sessions and 87.4 ± 6.2% of HIT sessions. Attendance decreased during the second 16-week phase of the study (85.2 ± 5.9% and 84.1 ± 5.0% of sessions for LMIT and HIT programs, respectively). Two subjects from the control group withdrawn from the trial. Four subjects from the LMIT program did not complete this program (66% of the sessions). However, 10 (50%) patients assigned to the HIT group attended less than 66% of the sessions. There were significant differences in compliance rate between the three study groups (*p* = 0.012), with significant differences between the LMIT and HIT groups (*p* = 0.047)

Two different intensity training programs with intervals of aerobic exercises were evaluated in hypertensive subjects already treated with antihypertensive medication and recruited in the primary care setting. The study included two separate phases of 1-h sessions, three times a week, over 12 and 16 weeks, respectively, with a resting period of 7 weeks overlapping vacation period. The effects of interrupting training activities on potential lifestyle changes, loss of improvement in cardiovascular parameters, or attendance to the second phase of the study have not been evaluated.

An interesting finding was a higher compliance with this training program in the LMIT group compared to the HIT group, in which half of the patients dropped out. Such observation could be indicating a lower adherence to the HIT program. Therefore, significant improvements in BP observed in the HIT group vs. slight improvements in the LMTI group were penalized by a higher withdrawal rate. In overweight and obese adults, it has been reported that high intensity interval training and moderate-intensity continuous training showed no differences in exercise adherence after eight weeks [19]. In relation to the optimal intensity of the exercise for inducing a sustained weight or fat-mass loss in overweight/obese people, HIT appears to induce superior improvements in aerobic fitness, but decreases adherence and results in the completion of less exercise [20]. In fact, response to physical exercise could determine adherence to a training program in direct association with the degree of unpleasant perception of the tasks performed, particularly when they are developed close to fatigue [21,22]. Therefore, a pleasant sensation related with physical exercise practice is reduced when its intensity is above the anaerobic threshold probably due to the accumulation of lactate in blood and musculoskeletal tissues, so that exceeding the anaerobic threshold could affect negatively pleasant sensations [23,24]. By contrast, individualized training prescription with progressive increase of intensity may be a critical factor to improve tolerance [25,26], particularly in community-based programs addressed to sensitive patients (e.g., advanced age, and under pharmacological treatment). In a randomized study in which sedentary young adults were assigned to six weeks of HIT or moderate continuous training, enjoyment for HIT increased with training whereas enjoyment for moderate continuous training remained constant and lower [27].

Improvement in BP levels during the first 12-week training phase of the study were not obtained, probably suggesting that such duration for a therapeutic training program could be inadequate to achieve the desired effects on hypertensive patients under pharmacological treatment. Other studies have also shown beneficial effects on BP for training periods over 12 weeks [28,29,30]. However, for the second extended training period of 16 weeks, HIT was more beneficial than LMIT in reducing BP. In a randomized study of 42 individuals with a baseline BP ≥ 130/85 mmHg, moderate intensity continuous exercise as compared with high intensity interval exercise did not modify any of the 24-h AMBP monitoring [31]. Moreover, HIT may favor advantageous adaptations of physiopathological variables that contribute to the development of hypertension. Therefore, HIT may be effective in reducing blood pressure, even in a lesser period of time, by increasing of apelin and NOx plasma levels [32]. All these effects together with reduced training time and duration as compared with moderate intensity continuous exercise may have important implications in the management of hypertension [8].

ABPM recording is a useful tool for the characterization of BP profiles by registering multiple daytime and night-time BP measures, which ensure a more detailed circadian monitoring and avoid diagnosis errors based on a single, isolated daytime measure [33]. Furthermore, registered information allows to estimate variables like 24-h MAP, and to make a detailed characterization of circadian patterns in BP, such as dipper-non-dipper, which has turned to be critical to evaluate cardiovascular morbidity and mortality and first choice tool for the prediction of hypertension-mediated organ damage [13]. Previous studies have postulated a beneficial influence of a regular aerobic activity on strengthening the effect of the SBP and pulse pressure drop at night (“dipping”) despite the lack of the influence upon the 24-h SBP [34]. In this study, BP modifications are found in both daytime and night-time BP registers. In fact, a drop of SBP, DBP, and systolic and diastolic loads was noticed during the second phase of the training program, after the resting period. Indeed, both intensities of training are capable to cause such decrease in BP variables, although HIT showed significantly better results for both circadian periods, allowing a reduction of beta-blockers medication in two patients following the prescription of their family physicians.

Ambulatory blood pressure (ABP) variability is a single independent indicator of cardiovascular risk [35]. High daytime SBP variability has been associated with the progression of early carotid atherosclerosis and significantly higher rate of cardiovascular morbid events [36]. Regular aerobic exercise can reduce BP but without a reduction in BP variability [37]. Such phenomenon is comparable to the effects of beta-blockers on BP and BP variability. Despite the lacking effect on BP variability, exercise should be routinely recommended to hypertensive patients as a basic lifestyle modification that potently reduces BP and elicits a multitude of further cardiovascular benefits [37]. In this study, ABP variability remains unaltered in both intervention groups during both day and night-time AMBP monitoring.

The present findings, however, should be interpreted taking into account the limitations of the study, particularly, the relatively small number of participants initially assigned to the three study groups, and the fact that the number of dropouts was higher in the HIT group. The reasons for a lower rate of compliance with the HIT modality is unknown, although other factors that may had an influence on compliance, such as age, female gender or BMI seem unlikely because significant differences in the distribution of these variables among the study groups were not observed. On the other hand, differences in the use of antihypertensive medication were not found. However, regarding the effect of HIT and LMIT on the study outcomes, similar results were obtained in the analysis of the PP and ITT populations. It should be noted that the training program included the combination of strength and endurance exercises, which have shown to produce greater cardiovascular benefits in hypertension than if endurance or strength training is performed alone [38,39]. However, the independent effect of strength or endurance alone on BP in this study cannot be specified. Finally, the pathophysiological mechanisms by which HIT regulates BP are numerous, involving effects on cardiorespiratory fitness, endothelial function and its markers, insulin sensitivity, autonomic nervous system activity, arterial stiffness, and blood glucose and lipoproteins [40], outside the scope of discussion of this study. However, additional analyses of parameters such as nitrogen oxides (NOx), apelin or specific lipoproteins and hormones would contribute to identify the underlying mechanisms by which exercise training improves BP.

## 4. Conclusions

In this pilot clinical study, primary care prescription of physical exercise in the framework of a community-based program appears to be a suitable adjunct therapy to control BP in the management of hypertension. The training intervention divided into two phases of 12 and 16 weeks, respectively, separated by a seven-week resting period, showed beneficial effects in reducing BP in hypertensive subjects under antihypertensive medication. In relation to the intensity of training, HIT was more effective than LMIT in the second phase of the study, but also was associated with a lower compliance rate. The mechanistic background of the positive effects of HIT merits further investigation. Intensity of training should be individually prescribed to improve tolerance to more high intensity exercises.

## Figures and Tables

**Table 1:** Main characteristics of the training program.

Block	Aim	Type	Exercises	Duration min	LMIT, Intensity %		HIT, Intensity %	
					Time	MHR	Time %	MHR
Warm up	Flexibility and mobility	Warm up	Deep inspirations, general stretching by muscular groups	4–8				
Aerobic #1	CV endurance	Intervals	Grapevines, boxing, kicks	22	38	60	38	85
Aerobic #2	CV endurance	Constant	Grapevines, kicks	22	20	55	20	80
Tone up muscles	Muscle strength	Series	Elbow flexion, abdominal contractions, squats	10	20	70	20	90
Strength and endurance	Muscle strength and CV endurance	Intervals	Elbow flexion, abdominal contractions, squats, side shoulder elevation, scissor	12	11	60	11	75
Race technique	Muscle strength and CV endurance	Intervals	Standing hamstring curl, side jumps, long steps, knee-up	5	5	60	5	80
Average intensity % MHR					61.55		82.8	

LMIT: low-moderate intensity training; HIT: high intensity training; MHR: maximum heart rate; CV: cardiovascular.

**Table 2:** Demographic and clinical characteristics of the 60 hypertensive subjects who were randomized and 44 who completed the study.

Variables	High Intensity Training Group			Low-Moderate Intensity Training Group			Control Group		
	Baseline (*n* = 20)	Study End (*n* = 10)	Dropout (*n* = 10)	Baseline (*n* = 20)	Study End (*n* = 16)	Dropout (*n* = 4)	Baseline (*n* = 20)	Study End (*n* = 18)	Dropout (*n* = 2)
Age, years	54.2 ± 7.8	53.5 ± 7.5	55.0 ± 8.4	55.5 ± 6.3	56.1 ± 6.4	53.5 ± 5.8	60.0 ± 7.4	59.3 ± 8.2	61.0 ± 4.2
*p* value	0.784			0.833			0.950		
BMI, kg/m^2^	30.1 ± 3.9	30.4 ± 4.2	28.6 ± 3.2	30.1 ± 3.9	30.4 ± 4.2	29.0 ± 2.5	27.9 ± 4.0	28.2 ± 4.3	29.0 ± 2.5
*p* value	0.995			0.835			0.882		
Lean body mass, kg	50.3 ± 8.2	48.9 ± 10.8	51.7 ± 4.7	47.9 ± 9.7	48.6 ± 10.1	45.1 ± 7.4	45.6 ± 8.4	45.1 ± 8.9	47.8 ± 7.1
*p* value	0.878			0.823			0.912		
Fat body mass, kg	27.5 ± 8.6	27.6 ± 9.3	26.6 ± 7.6	31.6 ± 9.2	32.7 ± 9.6	27.4 ± 6.3	27.3 ± 7.6	28.5 ± 7.5	21.2 ± 7.1
*p* value	0.979			0.716			0.746		
Systolic BP, mmHg	131.2 ± 9.1	131.5 ±12.3	131.0 ± 4.8	128.6 ± 7.7	128.6 ± 8.3	128.2 ± 5.7	126.7 ± 9.7	126.2 ±10.2	129.0 ± 9.9
*p* value	0.921			0.978			0.907		
Diastolic BP, mmHg	80.9 ± 6.0	81.3 ± 7.3	80.5 ± 4.6	79.7 ± 4.7	79.9 ± 4.4	78.8 ± 6.5	78.3 ± 9.2	77.7 ± 9.8	81.5 ± 6.3
*p* value	0.878			0.920			0.826		

Data are expressed as mean ± standard deviation; BMI: body mass index; BP: blood pressure; *p* value for within-group comparisons with the Mann-Whitney *U* test; analysis of variance (ANOVA) for repeated measures for between-group comparisons: *p* = 0.158 for age, *p* = 0.343 for BMI, *p* = 0.313 for fat body mass.

**Table 3:** Antihypertensive medication in the three study groups.

	Antihypertensive Pharmacological Treatment					
	ACE Inhibitors	ARB	α-Blockers	β-Blockers	Calcium Antagonists	Diuretics
Control group, *n* (%)	2 (10.0)	13 (65.0)	0(0)	3 (15.0)	5 (24.9)	5 (24.9)
LMIT, *n* (%)	2 (10.0)	15 (75.0)	0(0)	5 (24.9)	3 (15.0)	7 (35.0)
HIT, *n* (%)	2 (10.0)	9 (45.0)	2 (10.0)	3 (15.0)	0 (0)	3 (15.0)
Total, *n* (%)	6 (10.0)	37 (61.7)	2 (3.3)	11 (18.3)	8 (13.3)	15 (25.0)
*p* value	1.0	0.139	0.246	0.641	0.245	0.344

Data are reported as n (number) and % (percentage of the total for each group); ACE inhibitors: angiotensin-converting enzyme inhibitors; ARB: angiotensin receptor blockers; *p* value for between-group comparison with the chi-square test with each of these treatments.

**Table 4:** Summary of data from ABPM recordings overall 24 h.

Variable	Group	T1 (±SD)	Δ T2−T1 (95% CI)	T3 (±SD)	Δ T4−T3 (95% CI)	Δ T4−T1 (95% CI)	*P*_T2−T1_	*P*_T4−T3_	*P*_T4−T1_
Average SBP (mmHg)	Control	126.2 ± 10.2	0.2 (−3.7 to 4.1)	125.1± 9.4	1.4 (−3.2 to 6.0)	0.3 (−4.0 to 4.6)	0.860	**<0.001**	**<0.001**
	LMIT	128.7 ± 8.3	1.1(−2.0 to 4.2)	130.1 ± 10.4	−3.1 (−6.7 to 0.6)	−1.6 (−5.0 to 1.7)			
	HIT	131.5 ± 12.3	0.3 (–3.6 to 4.2)	134.0 ± 12.7	−10.8 (−15.4 to −6.2) *^†‡^	−8.3 (−12.6 to −4.0) *			
Average DBP (mmHg)	Control	77.7 ± 9.8	0.3 (−2.7 to 3.3)	78.7 ± 9.6	−0.3 (−2.8 to 2.1)	0.7 (−2.7 to 4.1)	0.611	**<0.001**	**<0.001**
	LMIT	79.9 ± 4.4	0.9 (−1.5 to 3.3)	81.3 ± 5.6	−2.4 (−4.4 to −0.5) *	−1.1 (−3.7 to 1.6)			
	HIT	81.3 ± 7.3	−0.5 (−3.5 to 2.5)	83.4 ± 7.0	−8.3 (−10.8 to −5.8) *^†‡^	−6.2(−9.6 to −2.8) *			
Average MAP (mmHg)	Control	93.9 ± 9.3	0.3 (−2.8 to 3.3)	94.2 ± 9.1	0.3 (−2.7 to 3.2)	0.6 (−2.8 to 3.9)	0.688	**<0.001**	**<0.001**
	LMIT	96.1 ± 4.7	0.9 (−1.4 to 3.3)	97.5 ± 6.8	−2.6 (−5.0 to −0.3) *	−1.3 (−3.9 to 1.4)			
	HIT	98.0 ± 7.4	−0.2 (−3.3 to 2.8)	100.3 ± 7.7	−9.1 (−12.1 to −6.2) *^†‡^	−6.9 (−10.2 to −3.5) *			
Average PP (mmHg)	Control	48.5 ± 7.5	−0.1 (−3.2 to 3.0)	48.4 ± 7.1	0.1 (−3.1 to 3.3)	−0.4 (−3.6 to 2.8)	0.838	0.248	0.658
	LMIT	48.8 ± 8.0	0.2 (−2.2 to 2.6)	49.0 ± 8.1	−0.6 (−3.1 to 1.8)	−0.6 (−3.1 to 2.0)			
	HIT	50.2 ± 12.0	0.8 (−2.3 to 3.9)	51.0 ± 12.3	−2.5 (−5.7 to 0.7)	−2.1 (−5.3 to 1.1)			
SD of SBP (mmHg)	Control	11.9 ± 3.2	−0.8 (−3.5 to 1.9)	11.9 ± 3.0	−1.0 (−3.3 to 1.3)	−1.1 (−3.5 to 1.4)	0.243	0.275	0.073
	LMIT	12.7 ± 3.3	−0.3 (−2.4 to 1.9)	12.3 ± 3.0	−0.8 (−2.6 to 1.0)	−1.2 (−3.2 to 0.8)			
	HIT	13.7 ± 2.4	1.4 (−1.3 to 4.1)	13.6 ± 3.8	−2.5 (−4.7 to −0.2) *	−2.6 (−5.0 to −0.1) *			
SD of DBP (mmHg)	Control	9.9 ± 2.5	−0.5 (−3.1 to 2.2)	9.5 ± 1.6	0.3 (−1.4 to 2.0)	−0.1 (−2.2 to 2.0)	0.267	0.077	0.146
	LMIT	11.2 ± 2.1	−1.1 (−3.2 to 1.0)	10.0 ± 2.4	−0.3 (−1.6 to 1.0)	−1.4 (−3.1 to 0.2)			
	HIT	11.1 ± 1.8	0.9 (−1.8 to 3.5)	11.4 ± 2.7	−1.7 (−3.3 to 0.1)	−1.3 (−3.4 to 0.8)			
Systolic load (%)	Control	35.1 ± 26.0	−2.4 (−10.2 to 5.4)	34.4 ± 25.9	−0.6 (−14.8 to 13.6)	−1.3 (−16.5 to 13.9)	0.504	**<0.05**	**<0.05**
	LMIT	44.3 ± 25.9	1.1 (−5.1 to 7.3)	45.4 ± 29.6	−6.2 (−17.4 to 5.0)	−5.2 (−17.2 to 6.8)			
	HIT	59.8 ± 28.9	−2.3 (−10.2 to 5.5)	59.7 ± 25.1	−27.0 (−41.2 to −12.8) *^†‡^	−27.0 (−42.2 to −11.9) *			
Diastolic load (%)	Control	36.3 ± 27.2	0.5 (−16.7 to 17.7)	35.0 ± 26.5	0.8 (−12.2 to 13.8)	−0.6 (−55.6 to 54.4)	0.334	**<0.05**	0.148
	LMIT	45.8 ±16.6	1.7 (−11.9 to 15.4)	50.4 ± 22.1	−8.6 (−19.0 to 1.6)	−4.1 (47.6 to 39.4)			
	HIT	88.4 ± 116.1	−9.6 (−26.8 to 7.7)	54.9 ± 21.5	−26.9 (−40.0 to −14.0) *^†‡^	−60.5 (−115.5 to −5.5) *			

ABPM; ambulatory blood pressure monitoring; CI: confidence interval; LMIT: low-moderate intensity training; HIT; high intensity training; SBP: systolic blood pressure; DBP: diastolic blood pressure; MAP: mean arterial pressure; PP: pulse pressure; Δ is the increment of blood pressure for the different periods of the study T1 (baseline) and T2 (end) of the first 12-week first phase of the study; T3 (baseline at week 19 after the 7-week resting period) and T4 (at week 35) at the end of the second 16-week phase of the study. Statistically significant values are in bold; * within-group significant increment (ANOVA for repeated measures group and time); ^†^ between-group significant increment (training vs. control groups); ^‡^ between-group significant increment (LMIT vs. HIT). Dropouts, *n* = 44, are excluded.

**Table 5:** Summary of data from ABPM recordings during daytime.

Variable	Group	T1 (±SD)	Δ T2−T1 (95% CI)	T3 (±SD)	Δ T4−T3 (95%CI)	Δ T4−T1 (95%CI)	*P*_T2−T1_	*P*_T4−T3_	*P*_T4−T1_
Average SBP (mmHg)	Control	128.1 ± 9.1	0.2 (−4.5 to 4.9)	127.9 ± 8.9	−0.2 (−5 to 4.5)	−0.4 (−5.2 to 4.4)	0.995	**<0.001**	**<0.001**
	LMIT	132.3 ± 8.2	0.0 (−3.7 to 3.7)	132.5 ± 10.1	−3.6 (−7.3 to 0.2)	−3.4 (−7.2 to 0.4)			
	HIT	134.7 ± 12.5	0.0 (−4.7 to 4.7)	137.2 ± 13.9	−11.2 (−16.0 to −6.4) *^†‡^	−8.7 (−13.5 to −0.4) *			
Average DBP (mmHg)	Control	79.6 ± 10.0	−0.2 (−4.4 to 4.0)	79.8 ± 10.0	−0.4 (−3.2 to 2.4)	−0.2 (−3.7 to 3.3)	0.720	**<0.001**	**<0.005**
	LMIT	83.4 ± 4.8	0.3 (−3.1 to 3.6)	83.9 ± 6.0	−2.4 (−4.6 to −0.3) *	−2.0 (−4.7 to 0.8)			
	HIT	84.5 ± 7.5	−1.3 (−5.5 to 2.9)	86.2 ± 7.5	−8.2 (−11.0 to −5.4) *^†‡^	−6.5 (−10.0 to −3.0) *			
Average MAP (mmHg)	Control	95.8 ± 9.0	−0.1 (−4.1 to 3.9)	95.8 ± 8.9	−0.3 (−3.6 to 2.9)	−0.3 (−3.9 to 3.3)			
	LMIT	99.7 ± 4.9	0.2 (−3.0 to 3.3)	100.1 ± 6.9	−2.8 (−5.4 to −0.2)*	−2.4 (−5.3 to 0.4)			
	HIT	101.2 ± 7.6	−0.9 (−4.9 to 3.1)	103.2 ± 8.4	−9.2 (−12.5 to −5.9) *^†‡^	−7.2 (−10.8 to −3.6) *			
Systolic load (%)	Control	31.2 ± 25.7	−1.7 (−17.7 to 14.4)	31.0 ± 25.7	−1.1 (−17.5 to 15.3)	−1.4 (−18.3 to 15.5)	0.335	**<0.05**	**<0.05**
	LMIT	40.1 ± 27.2	0.9 (−11.7 to 13.6)	40.7 ± 31.1	−8.7 (−21.6 to 4.3)	−8.1 (−21.4 to 5.3)			
	HIT	56.7 ± 28.4	−9.9 (−25.9 to 6.2)	53.6 ± 33.4	−24.4 (−40.8 to −8.0) *^†^	−27.5 (−44.4 to −10.6) *			
Diastolic load (%)	Control	26.9 ± 26.1	1.6 (−17.4 to 20.6)	27.3 ± 27.2	1.2 (−13.1 to 15.4)	1.6 (−16.2 to 19.4)	0.512	**<0.005**	**<0.05**
	LMIT	40.7 ± 18.6	1.4 (−13.6 to 16.4)	45.6 ± 23.1	−9.9 (−21.2 to 1.4)	−5 (−19.1 to 9.1)			
	HIT	48.8 ± 26.1	−7.8 (−26.8 to 11.2)	51.8 ± 25.7	−27.3 (−41.6 to −13.1) *^†‡^	−24.3 (−42.2 to −6.5) *			

ABPM; ambulatory blood pressure monitoring; CI: confidence interval; LMIT: low-moderate intensity training; HIT; high intensity training; SBP: systolic blood pressure; DBP: diastolic blood pressure; MAP: mean arterial pressure; Δ is the increment of blood pressure for the different periods of the study T1 (baseline) and T2 (end) of the first 12-week first phase of the study; T3 (baseline at week 19 after the 7-week resting period) and T4 (at week 35) at the end of the second 16-week phase of the study. Statistically significant values are in bold; * within-group significant increment (ANOVA for repeated measures group and time); ^†^ between-group significant increment (training vs. control groups); ^‡^ between-group significant increment (LMIT vs. HIT).

**Table 6:** Summary of data from ABPM recordings during night-time.

Variable	Group	T1 (±SD)	Δ T2−T1 (95% CI)	T3 (±SD)	Δ T4−T3 (95% CI)	Δ T4−T1 (95% CI)	*P*_T2−T1_	*P*_T4−T3_	*P*_T4−T1_
SBP decline (mmHg)	Control	6.2 ± 9.0	−0.7 (−8.5 to 7.1)	5.0 ± 8.6	−0.3 (−5.2 to 4.6)	−1.5 (−7.6 to 4.6)	0.635	0.816	0.936
	LMIT	11.4 ± 7.3	−2.4 (−8.5 to 3.8)	8.8 ± 7.7	−1.6 (−5.5 to 2.3)	−4.3 (−9.1 to 0.6)			
	HIT	10.6 ± 4.9	−0.5 (−8.3 to 7.3)	11.0 ± 7.7	−1.7 (−6.6 to 3.2)	−1.3 (−7.4 to 4.8)			
DBP decline (mmHg)	Control	7.0 ± 5.7	−1.6 (−9.2 to 6.0)	6.8 ± 6.1	−2.3 (−6.7 to 2.1)	−2.5 (−7.9 to 2.9)	0.991	0.561	0.960
	LMIT	10.9 ± 5.2	−1.7 (−7.8 to 4.3)	9.7 ± 5.6	−0.1 (−3.6 to 3.4)	−1.4 (−5.6 to 2.9)			
	HIT	10.8 ± 4.3	−2.1 (−9.7 to 5.5)	11.0 ± 5.7	−1.2 (−5.6 to 3.2)	−1.0 (−6.4 to 4.4)			
Average SBP (mmHg)	Control	121.9 ± 14.7	0.9 (−5.1 to 6.9)	122.9 ± 13.6	0.1 (−5.8 to 6.0)	1.1 (−5 to 7.2)	0.753	**<0.05**	**<0.05**
	LMIT	120.9 ± 10.2	2.4 (−2.3 to 7.1)	123.8 ± 12.9	−2.0 (−6.7 to 2.7)	0.9 (−4.0 to 5.7)			
	HIT	124.1 ± 11.8	0.5 (−0.5 to 6.5)	126.2 ± 10.4	−9.5 (15.4 to −3.6) *^†‡^	−7.4(−13.5 to −1.3) *			
Average DBP (mmHg)	Control	72.6 ± 11.0	1.4 (−3.7 to 6.5)	73.0 ± 10.7	1.9 (−2.5 to 6.3)	2.3 (−3.3 to 7.9)	0.871	**<0.001**	**<0.05**
	LMIT	72.4 ± 5.0	2.0 (−2.0 to 6.0)	74.3 ± (6.8)	−2.3 (−5.8 to 1.2)	−0.6 (−5.0 to 3.9)			
	HIT	73.7 ± 8.4	0.8 (−4.3 to 5.9)	75.2 ± 7.1	−7.0 (−11.4 to −2.6) *^†‡^	−5.5 (−11.1 to 0.1)			
Average MAP (mmHg)	Control	89.0 ± 11.7	1.2 (−3.9 to 6.3)	89.6 ± 11.1	1.3 (−3.2 to 5.8)	1.9 (−3.6 to 7.4)	0.818	**<0.05**	**<0.05**
	LMIT	88.6 ± 5.7	2.1 (−1.9 to 6.2)	90.7 ± 8.4	−2.2 (−5.8 to 1.3)	−0.1 (−4.4 to 4.2)			
	HIT	90.5 ± 8.0	1.7 (−2.5 to 5.9)	92.2 ± 7.2	−7.8 (−12.3 to −3.4) *^†‡^	−6.1 (−11.6 to −0.7) *			
Systolic load (%)	Control	45.5 ± 35.8	5.0 (−14.6 to 24.6)	47.9 ± 34.5	5.2 (−24.9 to 35.4)	7.6 (−13.2 to 28.4)	0.935	**<0.05**	0.151
	LMIT	53.7 ± 33.0	3.0 (−12.5 to 18.6)	59.4 ± 34.7	−3.0 (−26.9 to 20.8)	2.6 (−13.8 to 19.0)			
	HIT	58.5 ± 38.0	1.4 (−18.2 to 21.0)	77.9 ± 56.1	−35.9 (−66.0 to −5.7) *^†‡^	−16.5 (−37.3 to 4.3)			
Diastolic load (%)	Control	52.6 ± 29.3	2.0 (−15.4 to 19.4)	55.4 ± 29.4	2.9 (−14.2 to 20.1)	5.7 (−15.7 to 27.1)	0.687	**<0.05**	**<0.05**
	LMIT	57.6 ± 21.4	8.0 (−5.7 to 21.8)	64.7 ± 28.5	−7.5 (−21.1 to 6.0)	−0.4 (−17.3 to 16.5)			
	HIT	58.0 ± 24.5	2.6 (−14.8 to 20.0)	63.3 ± 17.2	−26.6 (−43.8 to −9.5) *^†‡^	−21.3 (−42.7 to 0.1)			

ABPM; ambulatory blood pressure monitoring; CI: confidence interval; LMIT: low-moderate intensity training; HIT; high intensity training; SBP: systolic blood pressure; DBP: diastolic blood pressure; MAP: mean arterial pressure; Δ is the increment of blood pressure for the different periods of the study T1 (baseline) and T2 (end) of the first 12-week first phase of the study; T3 (baseline at week 19 after the 7-week resting period) and T4 (at week 35) at the end of the second 16-week phase of the study. Statistically significant values are in bold; * within-group significant increment (ANOVA for repeated measures group and time); ^†^ between-group significant increment (training vs. control groups); ^‡^ between-group significant increment (LMIT vs. HIT).

## Data Availability

No new data were created or analyzed in this study. Data sharing is not applicable to this article.

## Supplementary Materials

The following are available online at https://www.mdpi.com/article/10.3390/jpm11040291/s1, Table S1: Summary of data from ABPM recordings overall 24 h. Intention to treat analysis.
