# Supplementary material for: High versus Low-Moderate Intensity Exercise Training Program as an Adjunct to Antihypertensive Medication: A Pilot Clinical Study
Source: J Pers Med. 2021 Apr 10;11(4):291. doi: 10.3390/jpm11040291 (PMC8069909; doi:10.3390/jpm11040291)
Supplement: Supplementary file 1 [file jpm-11-00291-s001.pdf]

Table S1. Summary of data from ABPM recordings overall 24 hours. Intention to treat analysis.

Variable	Group	T1 (± SD)	Δ T2 − T1 (95 % CI)	T3 (± SD)	Δ T4 − T3 (95% CI)	Δ T4 − T1 (95% CI)	P_{T2-T1}	P_{T4-T3}	P_{T4-T1}
Average SBP (mmHg)	Control	126.7 ± 9.7	0.3 (−3.0 to 3.5)	125.1 ± 9.0	2.8 (−1.7 to 7.3)	1.2 (−3.8 to 6.2)	0.396	**<0.001**	**<0.001**
	LMIT	128.6 ± 7.7	0.8 (−1.7 to 3.3)	129.9 ± 9.9	**−3.6 (−7.1 to −.01) ***	−2.3 (−6.2 to 1.6)			
	HIT	130.5 ± 8.9	−0.1 (−3.5 to 1.6)	132.9 ± 10.5	**−9.8 (−13.3 to −6.3) ***	**−7.4 (−11.2 to −3.5) ***			
Average DBP (mmHg)	Control	78.3 ± 9.2	0.2 (−2.2 to 2.5)	79.1 ± 8.9	0.2 (−2.5 to 2.8)	1.0 (−2.6 to 4.6)	0.720	**<0.001**	**<0.001**
	LMIT	79.6 ± 4.7	1.0 (−0.8 to 2.9)	80.6 ± 5.7	**−2.5 (−4.6 to −0.4) ***	−1.5 (−4.3 to 1.3)			
	HIT	80.9 ± 6.0	−0.8 (−2.6 to 1.0)	83.2 ± 6.0	**−7.7 (−9.8 to −5.7) ***	**−5.4 (−8.2 to −2.6) ***			
Average MAP (mmHg)	Control	94.4 ± 8.8	0.2 (−2.1 to 2.5)	94.4 ± 8.5	1.1 (−2.0 to 4.1)	1.0 (−2.8 to 4.9)	0.151	**<0.001**	**<0.001**
	LMIT	96.0 ± 4.8	1.0 (−0.8 to 2.8)	97.0 ± 6.7	**−2.8 (−5.2 to −0.5) ***	−1.8 (−4.8 to 1.2)			
	HIT	97.4 ± 5.8	−0.9 (−2.7 to 0.9)	99.8 ± 6.5	**−8.4 (−10.8 to −6.0) ***	**−6.1 (−9.1 to −3.1) ***			
Average PP (mmHg)	Control	48.3± 6.9	0.08 (−2.8 to 3.0)	47.8 ± 5.6	0.7 (−2.1 to 3.6)	−0.2 (−2.7 to 3.2)	0.969	**<0.05**	0.365
	LMIT	49.0 ± 7.3	−2.5 (−2.5 to 2.0)	49.0 ± 6.7	−0.8 (−3.0 to 1.4)	−0.8 (−3.1 to 1.5)			
	HIT	49.6 ± 8.8	−0.2 (−2.4 to 2.1)	50.4 ± 8.7	**−2.7 (−5.0 to −0.5) ***	−1.9 (−4.2 to 0.3)			
SD of SBP (mmHg)	Control	12.4 ± 3.3	−1.9 (−5.3 to 1.4)	11.4 ± 2.9	−1.0 (−3.4 to 1.5)	−2.0 (−4.8 to 1.0)	**<0.05**	0.243	**<0.001**
	LMIT	13.6 ± 4.3	−1.1 (−3.7 to 1.5)	12.5 ± 3.8	−0.9 (−2.8 to 1.0)	−2.0 (−4.2 to 0.3)			
	HIT	14.9 ± 4.3	−0.6 (−2.8 to 1.6)	14.3 ± 4.9	**−2.4 (−4.3 to −0.6) ***	**−3.0 (−5.3 to −0.8) ***			
SD of DBP (mmHg)	Control	9.9 ± 2.4	−1.5 (−4.5 to 1.5)	8.7 ± 2.3	0.2 (−2.2 to 2.5)	−1.0 (−4.2 to 2.1)	0.661	0.848	0.884
	LMIT	11.8 ± 2.6	−1.3 (−3.7 to 1.0)	10.2 ± 2.9	−0.4 (−2.3 to 1.4)	−2.0 (−4.5 to 0.4)			
	HIT	12.1 ± 2.5	−0.4 (−2.7 to 1.9)	11.0 ± 3.5	−0.1 (−2.0 to 1.7)	−1.3 (−3.7 to 1.1)			
Systolic load (%)	Control	35.7 ± 23.6	−1.8 (−8.2 to 4.6)	34.3 ± 23.6	1.4 (−11.9 to 14.7)	0.02 (−13.7 to 13.7)	0.335	**<0.05**	**<0.05**
	LMIT	41.8 ± 24.5	1.6 (−3.3 to 6.6)	43.5 ± 29.4	−6.2 (−16.5 to 4.1)	−4.5 (−15.1 to 6.1)			
	HIT	46.0 ± 26.5	1.9 (−6.8 to 10.5)	47.9 ± 28.8	**−20.5(−30.5 to −10.0) ***	**−18.4 (−29.0 to −7.8) ***			
Diastolic load (%)	Control	38.1 ± 25.0	0.3 (−13.7 to 14.4)	33.8 ± 24.3	10.0 (−7.3 to 27.3)	5.7 (−39.7 to 51.1)	0.687	**<0.05**	0.387
	LMIT	44.2 ± 15.6	2.5 (−8.4 to 13.3)	46.7 ± 24.2	−6.1 (−19.5 to 7.3)	−3.6 (−38.8 to 31.6)			
	HIT	59.9 ± 86.0	−2.9 (−13.8 to 8.0)	49. 1 ± 21.6	**−16.0 (−29.4 to −2.6) ***	−26.8 (−62.0 to 8.5)			
